# Highly efficient degradation of polybutylene succinate (PBS) and polycaprolactone (PCL) by a recombinant marine fungal cutinase

**DOI:** 10.1128/aem.00833-25

**Published:** 2025-08-14

**Authors:** Fengjuan Lang, Fan Fei, Chaomin Sun, Shimei Wu

**Affiliations:** 1 College of Life Sciences, Qingdao University 12593, Qingdao, China; 2 CAS and Shandong Province Key Laboratory of Experimental Marine Biology & Center of Deep Sea Research, Institute of Oceanology, Chinese Academy of Sciences 53014, Qingdao, China; 3 Laboratory for Marine Biology and Biotechnology, Qingdao Marine Science and Technology Center, Qingdao, China; 4 College of Earth Science, University of Chinese Academy of Sciences, Beijing, China; Shanghai Jiao Tong University, Shanghai, China

**Keywords:** polybutylene succinate, polycaprolactone, marine fungus, cutinase, enzymatic degradation

## Abstract

Biodegradable plastics, such as polybutylene succinate (PBS) and polycaprolactone (PCL), pose potential ecological risks due to their slow degradation rates in natural environments. Therefore, there is an urgent need to develop efficient enzymatic degradation technologies for the end-of-life management of PBS and PCL. In this study, we identified a marine fungal cutinase, *Aa*Cut10, which exhibits significant degradation activity on PBS and PCL films under mild conditions. *Aa*Cut10 can achieve degradation rates of 26.33% for PBS and 85.67% for PCL films within 20 min at 37°C, corresponding to degradation efficiencies of 315.96  kg PBS·(mol *Aa*Cut10·h)⁻¹ and 1028.04  kg PCL·(mol *Aa*Cut10·h)⁻¹, respectively. Notably, *Aa*Cut10 showed even higher degradation efficiency on PBS and PCL emulsions, achieving degradation rates of 81.88% for PBS and 99.45% for PCL after just 1 min at room temperature. Product analysis showed that *Aa*Cut10 degrades PBS into monomers and dimers and completely degrades PCL into monomers. The optimal degradation temperature for *Aa*Cut10 was 23°C for both PBS and PCL. Even at 4°C, *Aa*Cut10 retained high degradation activity, with relative activities of 60.58% for PBS emulsion and 81.41% for PCL emulsion. Additionally, Ca^2+^, Mg^2+^, and Mn^2+^ significantly enhanced the degradation activity of *Aa*Cut10, and the enzyme remained stable in the presence of chemicals such as methanol, ethanol, and glycerol. Finally, we identified that the amino acids Leu209 and Leu216 in *Aa*Cut10 play key roles in its degradation functions toward both PBS and PCL.

**IMPORTANCE:**

Although biodegradable plastics can be degraded, their degradation rates in natural environments are slower than expected. To address this issue, we identified a marine fungal cutinase, *Aa*Cut10, which efficiently degrades various polyesters, including PBS and PCL, into their corresponding monomers, thereby facilitating subsequent recycling and remanufacturing. Notably, *Aa*Cut10 achieves maximum degradation efficiency at ambient temperature (23°C) and retains high activity even at 4°C, meeting the energy efficiency requirements of industrial applications. Furthermore, *Aa*Cut10 exhibits high stability in the presence of chemicals, making it suitable for multiphase catalytic systems while the enhancing effects of metal ions on its activity provide tunable targets for process optimization. The catalytic properties of *Aa*Cut10 not only establish a theoretical framework for designing high-performance degrading enzymes but also offer essential support for developing eco-friendly plastic recycling systems.

## INTRODUCTION

Our planet is facing a severe plastic pollution crisis (1). Biodegradable plastics, which can be degraded and re-enter the biogeochemical cycle, have emerged as a promising solution to this problem (2). In recent years, driven by market demand and policies, the production of biodegradable plastics has steadily increased, leading the industry to develop at an unprecedented pace (3). Commonly used biodegradable plastics include poly(butylene adipate-*co*-terephthalate) (PBAT), polylactic acid (PLA), polybutylene succinate (PBS), polycaprolactone (PCL), and polyhydroxybutyrate (PHB) (4).

PBS, a biodegradable polymer first introduced in 1993, remains widely used in industry today. Its excellent properties have enabled its broad application in fields such as agricultural films, compostable bags, non-woven fabrics, and surgical sutures (5–9). Additionally, PBS holds significant potential for single-use food packaging (10). In recent years, PCL has also gained considerable attention due to its low melting point, low viscosity, excellent thermal processability, biodegradability, and low toxicity (11–13). Currently, PCL is increasingly being used in compostable bags and bottles within the food industry (13, 14). In agriculture, PCL serves as a carrier for fertilizers, herbicides, and agricultural mulch films (15, 16). Despite their biodegradable properties, both PBS and PCL degrade slowly in natural environments. For example, studies have shown that less than 5% of PBS degrades after 10 weeks in seawater, whereas the weight of PCL films decreases by only approximately 13% after 38 weeks in sandy soil (17, 18). To prevent the large-scale accumulation of biodegradable plastics in the environment, it is essential to develop effective methods, such as enzymes with high degradation activity, to accelerate the indoor degradation of these polymers and establish an efficient end-of-life management system.

Microorganisms and their secreted hydrolases play a key role in the degradation of biodegradable plastics (19–22). Currently, the identified polyester-degrading enzymes can be mainly categorized into four classes: cutinases, which are primarily derived from fungi (23), such as *Fusarium* (24, 25), *Paraphoma chrysanthemicola* (26), and *Humicola insolens* (27); lipases, represented by *Candida antarctica* lipase B (CALB) (28); carboxylesterases, such as esterase HP from *Pseudomonas mendocina* (29); and proteases with esterase activity, for example, the serine protease from *Laceyella sacchari*, which shows a certain degree of degradation activity toward PBS, PLA, and other polymers (30). These enzymes depolymerize polyesters by attacking the ester bonds in the polymer backbone (31).

To enhance the performance of polyester-degrading enzymes and overcome bottlenecks such as limited degradation efficiency and poor stability, researchers are actively employing a variety of strategies, including protein engineering and the discovery of novel enzymes (32, 33). For example, the best mutant DepoPETase obtained through directed evolution and high-throughput screening showed a 1,407-fold increase in degradation product yield of amorphous polyethylene terephthalate (PET) films at 50 °C compared with the wild-type enzyme, along with a 23.3°C increase in melting temperature (34). In addition, omics technologies such as metagenomics and proteomics have provided powerful support for the efficient discovery of novel plastic-degrading enzymes (35). In terms of industrial application, the French company Carbios has successfully achieved large-scale enzymatic recycling of PET using engineered cutinases (36), marking a key step toward the commercialization of enzyme-based plastic recycling technology. Nevertheless, efficient enzymes capable of degrading polyesters such as PBS and PCL remain extremely scarce, and the development of high-performance enzymatic preparations continues to be a critical area of research.

In our previous study, we reported that two marine fungal cutinases, *Aa*Cut4 and *Aa*Cut10, exhibit high degradation activity toward the biodegradable plastic PBAT (37). In this study, we further explored and evaluated the degradation potential of *Aa*Cut4 and *Aa*Cut10 on PBS and PCL films and emulsions. Given the superior degradation efficiency of *Aa*Cut10, we focused on analyzing its degradation performance on PBS and PCL films and emulsions under various conditions. Additionally, we investigated the key amino acids in the active site of *Aa*Cut10 that contribute to its degradation activity.

## RESULTS

### Comparative analysis of degradation efficiencies of fungal cutinases *Aa*Cut10 and *Aa*Cut4 on PBS and PCL

**Fig 1:**
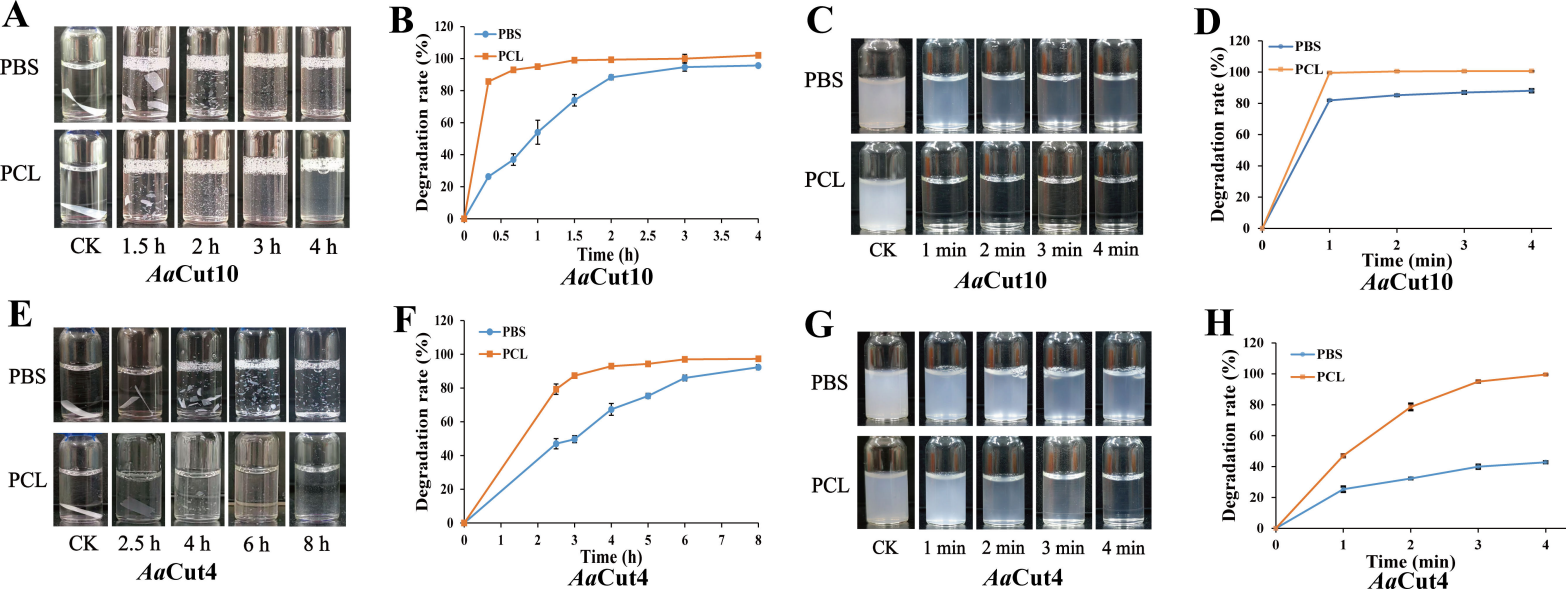
Degradation assays of PBS and PCL by fungal cutinases *Aa*Cut10 and *Aa*Cut4. (**A, B**) Degradation of PBS and PCL films by *Aa*Cut10 at different time points (**A**) and the corresponding degradation rate curves (**B**). (**C, D**) Degradation of PBS and PCL emulsions by *Aa*Cut10 at different time points (**C**) and the corresponding degradation rate curves (**D**). (**E, F**) Degradation of PBS and PCL films by *Aa*Cut4 at different time points (**E**) and the corresponding degradation rate curves (**F**). (**G, H**) Degradation of PBS and PCL emulsions by *Aa*Cut4 at different time points (**G**) and the corresponding degradation rate curves (**H**). “CK” denotes the negative control group, in which the enzyme solution is replaced by an equal volume of 50 mM Tris-HCl buffer (pH 8.0). The control images correspond to the time point with the longest incubation period for each respective experimental group. All measurements were performed in triplicate, and the results are presented as mean ± standard deviation (*n* = 3).

Since cutinases *Aa*Cut10 and *Aa*Cut4 from the marine fungus *Alternaria alternata* FB1 have been shown to efficiently degrade PBAT plastic in our previous study (37), we further evaluated their ability to degrade PBS and PCL. The results demonstrated that both enzymes exhibit high degradation activity toward PBS and PCL. When PBS and PCL films were incubated with *Aa*Cut10 at 37°C, the films were fragmented within 2 h, and the white powder became nearly invisible after 4 h. *Aa*Cut10 exhibited even higher degradation efficiency for PCL films compared with PBS (Fig. 1A). Notably, after incubation with *Aa*Cut10 for 20 min, PBS and PCL films exhibited weight losses of 26.33% and 85.67%, respectively, corresponding to degradation efficiencies of 315.96  kg PBS·(mol *Aa*Cut10·h)⁻¹ and 1028.04  kg PCL·(mol *Aa*Cut10·h)⁻¹. Following 2 h of treatment, degradation rates of the films reached 88.33% for PBS and 99.33% for PCL (Fig. 1B). Moreover, the degradation efficiency of *Aa*Cut10 significantly improved when PBS and PCL were emulsified. For PBS emulsion, *Aa*Cut10 achieved a degradation rate of 81.88% within 1 min at room temperature (23°C), and the emulsion became noticeably clearer after 4 min. In contrast, the PCL emulsion became completely transparent within 1 min of *Aa*Cut10 treatment, corresponding to a degradation rate of 99.45% (Fig. 1C and D).

To investigate the underlying cause of the rapid weight loss of PBS and PCL films treated with *Aa*Cut10, we evaluated their degradation behavior through macroscopic observation and microscopic structural analysis. After 20 min of incubation, irregular degradation marks appeared at the edges of the PCL film, whereas the overall structures of both films remained largely intact. Microscopic observation further revealed slight surface erosion and a few micropores on the PBS film. In contrast, the PCL film showed extensive damage—its “scaly” structure was almost completely eroded, and large cavities (~50 µm) had formed (Fig. S1). This pronounced microstructural destruction is consistent with the higher degradation rate of PCL, indicating that *Aa*Cut10 exhibits stronger degradative activity toward PCL.

Compared with *Aa*Cut10, *Aa*Cut4 exhibited weaker degradation activity toward PBS and PCL. After 4 h of incubation with *Aa*Cut4, visible fragmentation of the polymer films was observed, with degradation rates of 67.33% for PBS and 93.00% for PCL. However, even after 8 h of incubation, small fragments of the PBS film still remained, and the degradation rate reached 92.33% (Fig. 1E and F). Although the degradation efficiency of *Aa*Cut4 improved with emulsified substrates, it remained lower than that of *Aa*Cut10. After 4 min of incubation, *Aa*Cut4 achieved degradation rates of 42.75% for PBS emulsion and 99.48% for PCL emulsion, with the PBS emulsion remaining relatively turbid at this stage (Fig. 1G and H). Due to its superior degradation performance on both PBS and PCL, *Aa*Cut10 was selected as the primary focus of this study.

### Identification of PBS and PCL degradation products by cutinase *Aa*Cut10

**Fig 2:**
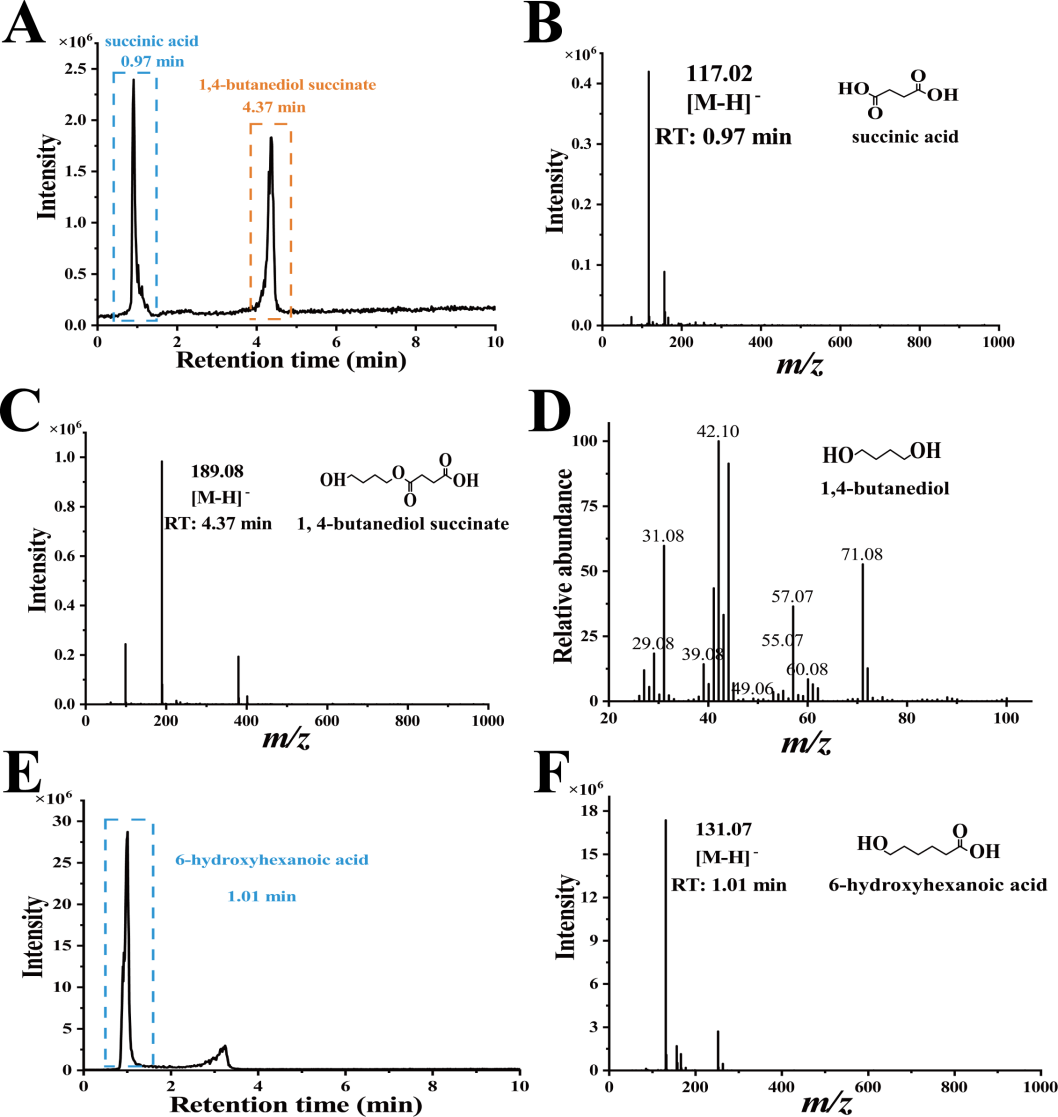
Analysis of degradation products of PBS and PCL by *Aa*Cut10. (**A–C**) HPLC-MS analysis of PBS film degradation products (succinic acid and 1,4-butanediol succinate) after 24 h of treatment with *Aa*Cut10: full-scan chromatogram (**A**), and ion chromatograms with corresponding chemical structures at retention times of 0.97 min (**B**) and 4.37 min (**C**). (**D**) Spectrum and structure of the degradation product (1,4-butanediol) from PBS film analyzed by GC-MS after incubation with *Aa*Cut10 for 24 h. (**E, F**) HPLC-MS analysis of PCL film degradation product (6-hydroxyhexanoic acid) after 24 h of treatment with *Aa*Cut10: full-scan chromatogram (**E**) and ion chromatograms with corresponding chemical structures at retention times of 1.01 min (**F**).

The analysis of degradation products not only reflects the enzyme’s degradation performance but also provides critical insights into subsequent application studies. Therefore, we analyzed the degradation products of PBS and PCL films after 24 h of incubation with *Aa*Cut10. For PBS, HPLC-MS analysis revealed two distinct chromatographic peaks at retention times (RT) of 0.97 min and 4.37 min (Fig. 2A). The corresponding mass spectra showed ion peaks at mass-to-charge ratios (*m/z*) of 117.02 and 189.08, which correspond to the PBS monomer (succinic acid) and PBS dimer (1,4-butanediol succinate), respectively (Fig. 2B and C). Considering that PBS is synthesized through the condensation of succinic acid and 1,4-butanediol (38, 39), the absence of 1,4-butanediol in the HPLC-MS profile is likely attributable to the method’s limited detection sensitivity for this compound, as previously reported (26). To confirm the presence of 1,4-butanediol, GC-MS analysis was conducted. This analysis successfully identified 1,4-butanediol (Fig. 2D), indicating that PBS films were hydrolyzed into succinic acid monomers, 1,4-butanediol monomers, and 1,4-butanediol succinate dimers.

HPLC-MS analysis of PCL degradation products yielded a single prominent chromatographic peak at RT of 1.01 min. The mass spectrum of this peak showed a predominant ion at *m/z* 131.07, corresponding to the PCL monomer (6-hydroxyhexanoic acid), and no polymeric forms were detected in the products (Fig. 2E and F). Collectively, it can be concluded that *Aa*Cut10 achieves relatively thorough degradation of both PBS and PCL. In contrast, most other studies have reported the presence of varying amounts of polymeric forms in degradation products after treatment with different enzymes (25, 28, 29).

### Effects of temperature and pH on the degradation activity of *Aa*Cut10

**Fig 3:**
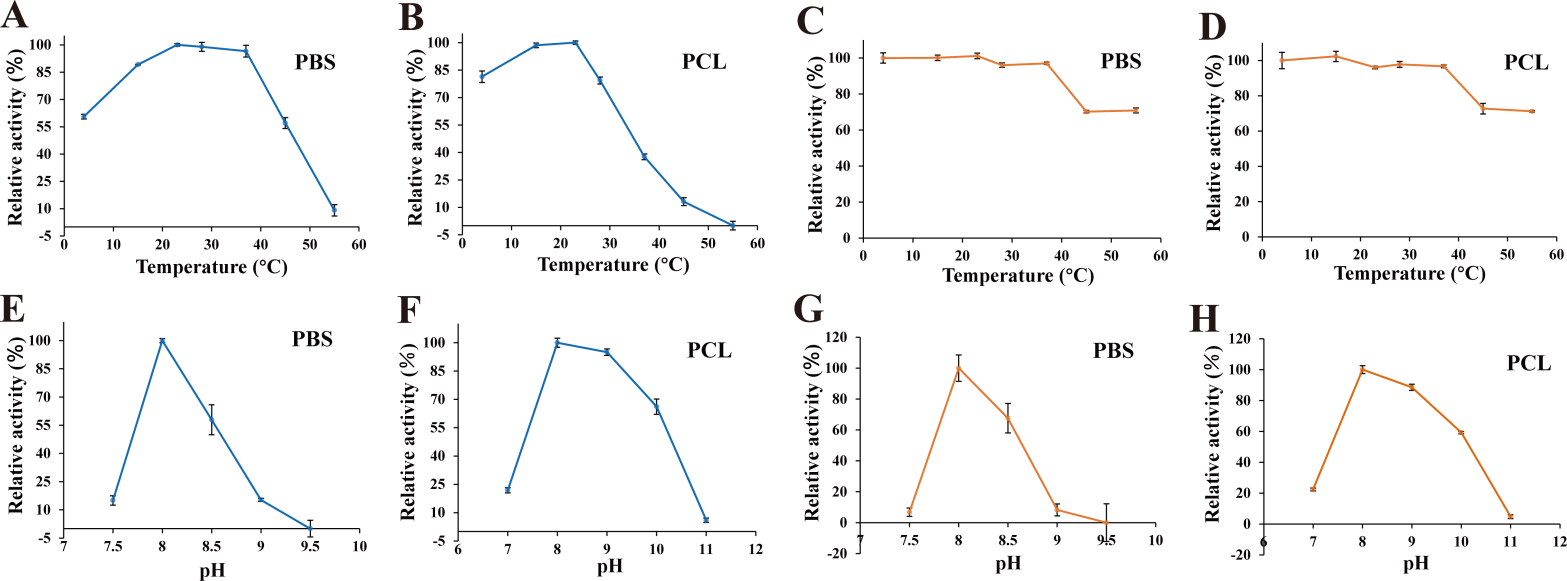
Effects of temperature and pH on the degradation activity of *Aa*Cut10. (**A, B**) Effects of temperature on the degradation activity of *Aa*Cut10 toward PBS and PCL emulsions. (**C, D**) Effects of temperature on the stability of *Aa*Cut10 toward PBS and PCL emulsions. (**E, F**) Effects of pH on the degradation activity of *Aa*Cut10 toward PBS and PCL emulsions. (**G, H**) Effects of pH on the stability of *Aa*Cut10 toward PBS and PCL emulsions. In the assay, 1 µM or 0.5 µM of *Aa*Cut10 was mixed with 2 mg/mL PBS or PCL emulsion at a 1:3 ratio in a 1 mL system. All measurements were performed in triplicate, and the results are presented as mean ± standard deviation (*n* = 3).

To identify factors influencing the degradation activity of *Aa*Cut10, we conducted a series of experiments. First, we evaluated the effect of temperature on *Aa*Cut10 activity across a range of 4°C–55°C. The results revealed that the optimal degradation temperature for *Aa*Cut10 was 23°C for both PBS and PCL emulsions. Notably, *Aa*Cut10 maintained high degradation activity even at low temperatures, retaining 60.58% activity on PBS emulsion and 81.41% on PCL emulsion at 4°C (Fig. 3A and B). Additionally, we assessed the temperature stability of *Aa*Cut10 by measuring its residual activity after incubation at different temperatures for 2 h. The results demonstrated that *Aa*Cut10’s degradation activity remained stable across the tested temperature range (4°C–55°C). Even after incubation at 55°C for 2 h, *Aa*Cut10 retained over 70% activity on both PBS and PCL (Fig. 3C and D). These findings indicate that *Aa*Cut10 exhibits excellent stability across a broad temperature range (4°C–55°C). Based on the robust low-temperature degradation activity of *Aa*Cut10 observed in emulsified systems, we further investigated its ability to degrade PBS and PCL films under similar conditions. After 4 h of incubation, *Aa*Cut10 demonstrated significantly higher degradation efficiency toward PCL across all tested temperatures. Notably, the degradation rates of PCL films exceeded 97% at 23°C, 28°C, and 37°C, whereas a substantial degradation of 75.67% was still achieved at 15°C (Fig. S2), highlighting its exceptional catalytic performance and broad temperature tolerance in PCL depolymerization.

The effect of pH on the degradation activity of *Aa*Cut10 toward PBS and PCL was also investigated. The results demonstrated that *Aa*Cut10 exhibited the highest degradation activity on both PBS and PCL at pH 8.0 while retaining over 65% activity on PCL even at pH 10.0. However, *Aa*Cut10 displayed higher pH sensitivity when PBS was used as the substrate, resulting in a narrower pH range for optimal activity (Fig. 3E and F). pH stability tests further revealed that the residual degradation activity of *Aa*Cut10 on PBS emulsion dropped to less than 10% at pH 9.0, whereas it remained around 90% on PCL emulsion under the same conditions (Fig. 3G and H). These findings indicate that *Aa*Cut10 maintains high degradation activity and stability on PCL across a relatively broad pH range.

### Effects of metal ions and chemicals on the degradation activity of *Aa*Cut10

**Fig 4:**
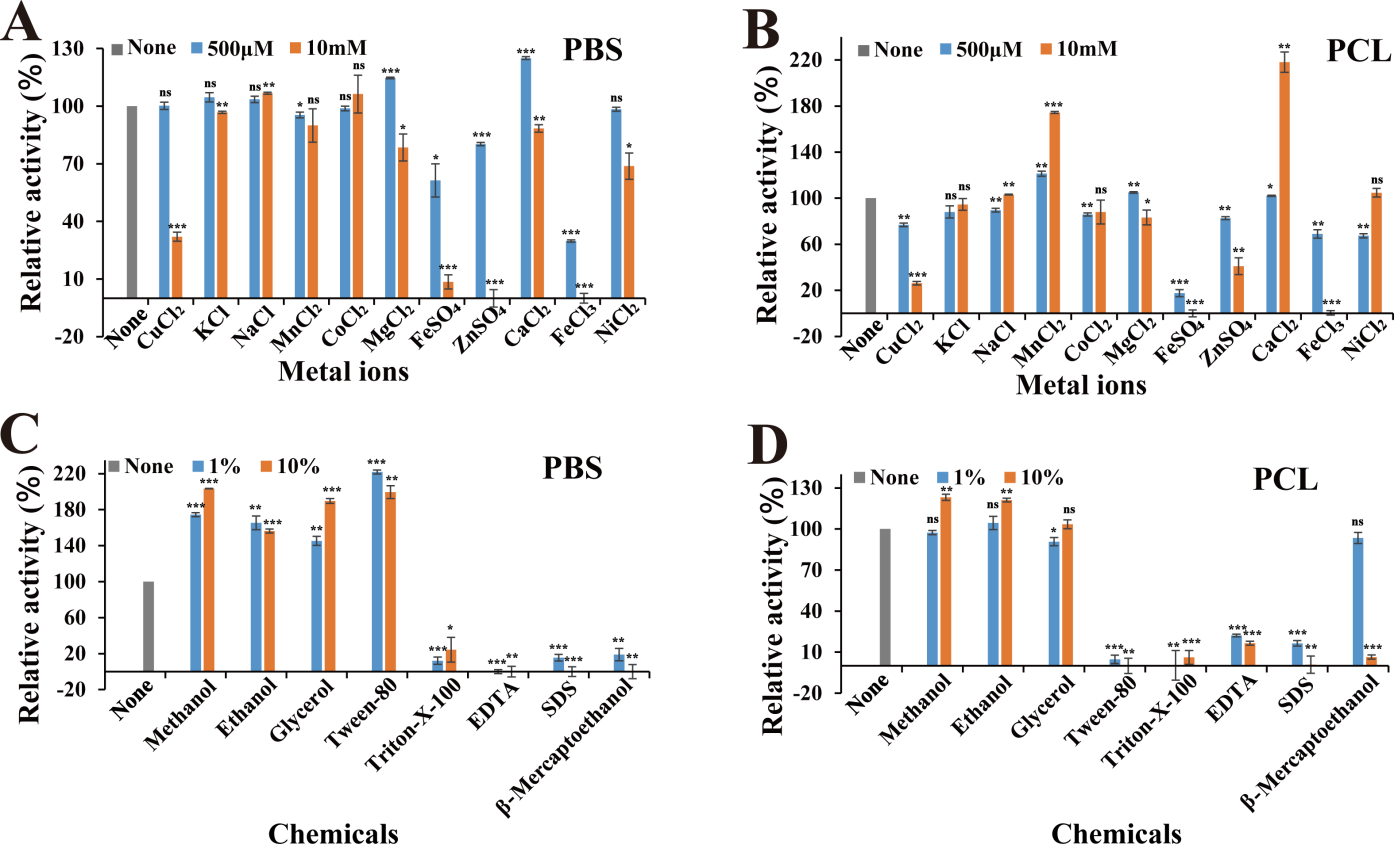
Effects of metal ions and chemicals on the degradation activity of *Aa*Cut10. (**A, B**) Effects of metal ions on the degradation activity of *Aa*Cut10 toward PBS and PCL emulsions. (**C, D**) Effects of chemicals on the degradation activity of *Aa*Cut10 toward PBS and PCL emulsions. In the assay, 1 µM or 0.5 µM of *Aa*Cut10 was mixed with 2 mg/mL PBS or PCL emulsion at a 1:3 ratio in a 1 mL system. “None” represents the absence of additional metal ions or chemicals. All measurements were performed in triplicate, and the results are presented as mean ± standard deviation (*n* = 3). Differences were considered statistically significant at *P* ≤ 0.05 (*P ≤ 0.05, **P ≤ 0.01, ***P ≤ 0.001 vs “None”). “ns” indicates no significance.

To investigate the effects of metal ions and chemicals on enzyme activity, the degradation capability of *Aa*Cut10 was assessed in PBS and PCL emulsions containing various metal ions and chemicals. The results revealed that *Aa*Cut10 is compatible with most metal ions. Specifically, 500 µM of Ca^2+^ and Mg^2+^ slightly enhanced *Aa*Cut10’s degradation activity in PBS emulsion, whereas 10 mM of Ca^2+^ and Mn^2+^ nearly doubled its activity in PCL emulsion. Notably, Ca^2+^ consistently enhanced *Aa*Cut10’s activity on both substrates, consistent with previous findings that *Aa*Cut10 contains a Ca^2+^-binding site (37). In contrast, 10 mM of Cu^2+^, Fe^2+^, Zn^2+^, and 500 µM or 10 mM of Fe^3+^ strongly inhibited *Aa*Cut10’s degradation activity in PBS, reducing it to less than 35%, with some ions completely abolishing activity. For PCL emulsion, 10 mM of Cu^2+^, Fe^3+^, and 500 µM or 10 mM of Fe^2+^ exhibited even stronger inhibition, reducing *Aa*Cut10’s degradation activity to below 30% (Fig. 4A and B). To assess its environmental applicability, we examined the degradation performance of *Aa*Cut10 on PBS and PCL films in a seawater environment. After 4 h of incubation in seawater, the degradation rates of PBS and PCL reached 31.00% and 60.67%, respectively (Fig. S3). These findings demonstrate that *Aa*Cut10 retains considerable degradation activity in a marine environment, particularly exhibiting effective degradation of PCL. This is consistent with the characteristics of *Aa*Cut10 as a marine-derived cutinase. Previous studies have indicated that marine-derived *A. alternata* also possesses some capacity to degrade PCL; however, these investigations are mostly preliminary and lack systematic analysis (40). Our study provides more in-depth evidence of the high degradation efficiency of *Aa*Cut10, thereby reinforcing its potential for application in practical environmental settings.

The effects of various chemicals on the degradation activity of *Aa*Cut10 were further investigated. The results demonstrated that Triton-X-100, EDTA, SDS, and β-mercaptoethanol significantly inhibited *Aa*Cut10’s activity on both PBS and PCL substrates, although 1% β-mercaptoethanol had no significant effect on PCL degradation (Fig. 4C and D). EDTA’s strong inhibition of *Aa*Cut10’s activity on both PBS and PCL aligns with its ability to chelate metal ions, further supporting that *Aa*Cut10 is a metal ion-dependent enzyme (41). Interestingly, certain chemicals positively influenced *Aa*Cut10’s activity. Methanol, ethanol, glycerol, and Tween-80 significantly enhanced its degradation activity on PBS by approximately two-fold. However, their effects on PCL were less pronounced, and Tween-80 nearly abolished *Aa*Cut10’s activity on PCL (Fig. 4C and D). The stability of *Aa*Cut10 in the presence of various metal ions and chemicals highlights its potential for diverse industrial applications in environmental systems.

### Orthogonal design-based analysis of factors influencing *Aa*Cut10-mediated degradation of PBS and PCL

To further analyze the effects of temperature, pH, and metal ions on the degradation performance of *Aa*Cut10, a three-factor, three-level orthogonal experiment was designed, and range analysis was conducted. The average relative degradation activities (k) of *Aa*Cut10 on PBS and PCL, as well as their ranges (R) under different factor levels, are summarized in Tables S1 and S2. The results showed that temperature (R = 38.91) had the most significant impact on *Aa*Cut10’s degradation activity toward PBS, whereas metal ion type (R = 52.14) was the dominant factor in PCL degradation. In contrast, metal ions (R = 15.35) and temperature (R = 9.96) had the least impact on *Aa*Cut10 degradation of PBS and PCL, respectively. Based on the analysis of k values at various factor levels, the optimal reaction conditions for *Aa*Cut10 degrading both PBS and PCL were inferred to be 23°C, pH 8.0, and Ca^2+^ (500 µM for PBS and 10 mM for PCL) (Table S1 and S2). These findings provide a theoretical basis for optimizing the application conditions of *Aa*Cut10 in PBS and PCL degradation.

### Identification of key amino acids determining *Aa*Cut10’s degradation activity toward PBS and PCL

**Fig 5:**
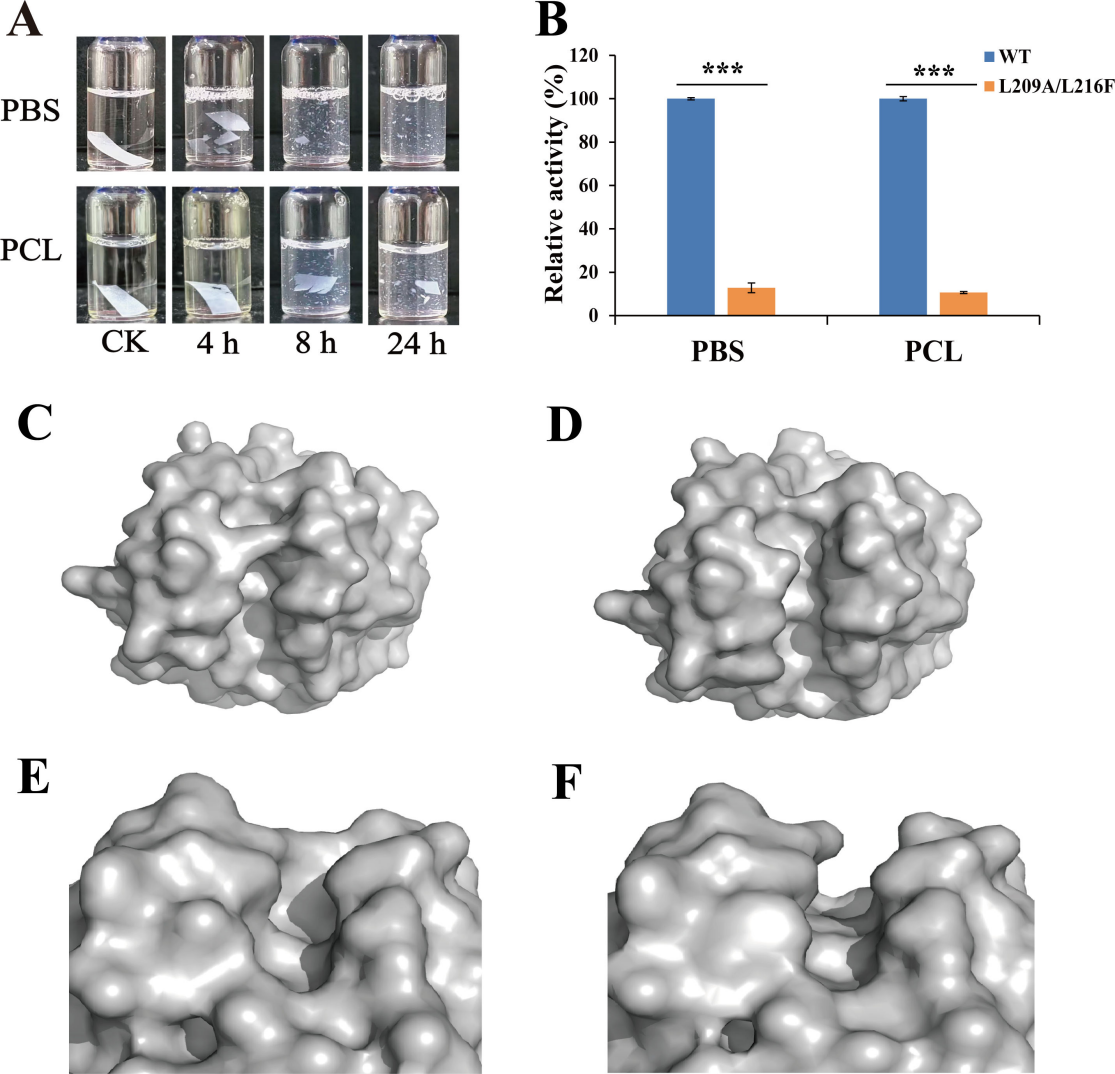
Comparison of PBS and PCL degradation rates and predicted structures of wild-type *Aa*Cut10 and its mutant L209A/L216F. (**A**) Degradation effects of PBS and PCL films (0.5 × 1 cm, ~2 mg) by the *Aa*Cut10 mutant L209A/L216F at different time intervals. “CK” denotes the negative control group, in which the enzyme solution is replaced by an equal volume of 50 mM Tris-HCl buffer (pH 8.0). The control images correspond to the time point with the longest incubation period for each respective experimental group. (**B**) Comparison of degradation activities of wild-type *Aa*Cut10 and its mutant L209A/L216F on PBS and PCL emulsions. In the assay, 2 µM or 1 µM of *Aa*Cut10 (or its mutant L209A/L216F) was mixed with 2 mg/mL PBS or PCL emulsion at a 1:3 ratio in a 1 mL system. All measurements were performed in triplicate, and the results are presented as mean ± standard deviation (*n* = 3). Differences were considered statistically significant at *P* ≤ 0.05 (***P ≤ 0.001). (**C, D**) Structural prediction showing changes in the active-site channel length between wild-type *Aa*Cut10 (**C**) and the L209A/L216F mutant (**D**). (**E, F**) Predicted structural changes at the active-site entrance between wild-type *Aa*Cut10 (**E**) and the L209A/L216F mutant (**F**).

To identify the amino acid residues critical for *Aa*Cut10’s degradation of PBS and PCL, site-directed mutagenesis was performed based on our previous study (37). Specifically, two hydrophobic residues located in the active site channel, Leu209 and Leu216, were mutated to Ala and Phe, respectively, in order to investigate the impact of structural changes in the catalytic channel on enzymatic degradation performance. Comparative degradation assays revealed that the L209A/L216F double mutant exhibited markedly reduced catalytic efficiency toward both PBS and PCL films relative to the wild-type enzyme. When incubated with the mutant, PBS films were fragmented into various sizes after 4 h, whereas PCL films showed only slight degradation. Even after 24 h, the L209A/L216F mutant failed to completely degrade PCL films (Fig. 5A). In contrast, wild-type *Aa*Cut10 nearly fully degraded both PBS and PCL films within 4 h (Fig. 1A). For the emulsion form, the L209A/L216F mutant retained less than 15% of the degradation activity on PBS and PCL compared with the wild-type (Fig. 5B). These results indicate that the Leu209 and Leu216 residues are essential structural elements for maintaining the degradation activity of *Aa*Cut10 and are critical determinants of its broad-spectrum degradation capability toward various polyester substrates.

To further elucidate the critical roles of Leu209 and Leu216 in the enzyme structure, we performed structural modeling and comparative analysis of wild-type *Aa*Cut10 and its L209A/L216F double mutant. The results revealed that from the perspective of the Leu209 mutation site, the substrate-binding pathway within the catalytic channel is extended in the double mutant (Fig. 5C and D). Meanwhile, the mutation at Leu216 to a bulkier Phe residue leads to a marked narrowing of the channel entrance (Fig. 5E and F). These structural perturbations observed from different mutation sites further highlight the synergistic roles and essential functions of Leu209 and Leu216 in maintaining the spatial configuration of the substrate-binding channel.

## DISCUSSION

In this study, we evaluated the degradation activity of *Aa*Cut10 toward PBS and PCL. After 20 min of enzymatic treatment, the degradation rates reached 26.33% for PBS and 85.67% for PCL, accompanied by pronounced surface erosion. Notably, large pore structures were observed on the surface of PCL films (Fig. 1B and Fig. S1). These morphological changes suggest that *Aa*Cut10 initiates degradation through surface erosion, which subsequently progresses to deeper perforation and eventual disintegration of the polymer matrix. This sequential degradation process accounts for the rapid macroscopic transition from an intact film to fragmented debris observed within a relatively short time frame. Notably, the degradation rates on PBS and PCL emulsions were significantly enhanced, reaching 81.88% and 99.45% within 1 min, respectively (Fig. 1D). The increased efficiency in emulsion form is likely due to the dispersion of polyester particles, which increases the surface area and collision probability between the enzyme and substrate, thereby accelerating degradation.

Synthetic polymers exhibit high persistence in natural environments, leading to the accumulation of plastic waste and exacerbating environmental pollution (42, 43). Recycling plastic waste is crucial for mitigating pollution and enhancing resource utilization, with the post-use value of biodegradable materials being a key consideration for future applications (44–46). Enzymatic degradation offers a sustainable approach, as the resulting monomers can be repurposed to synthesize new polymers or serve as raw materials for other valuable compounds (47). For instance, *Candida antarctica* lipase B degrades PCL into 6-hydroxyhexanoic acid monomers, which can be further upcycled into adipic acid—a versatile six-carbon chemical—using recombinant *Escherichia coli* (48). Our study demonstrated that *Aa*Cut10 is capable of converting PBS films into two corresponding monomers and a dimer and completely degrading PCL films into monomers (Fig. 2). These monomeric products are recyclable and can serve as raw materials for industrial production. These results highlight *Aa*Cut10’s significant potential for the degradation and recycling of biodegradable plastics, offering a promising solution for sustainable plastic waste management.

To highlight the significant advantages of *Aa*Cut10 in polyester degradation and its potential industrial applications, we conducted a comparative analysis with previously reported PBS- and PCL-degrading enzymes. Among these, the recombinant cutinase SiCut1 is recognized for its high polyester degradation activity. SiCut1 can achieve degradation rates of 98.0% for PCL and 12.1% for PBS within 48 h (49), with corresponding mass degradation efficiencies of 51.04  kg PCL·(mol SiCut1·h)⁻¹ and 3.78  kg PBS·(mol SiCut1·h)⁻¹. In contrast, *Aa*Cut10 exhibits markedly higher catalytic efficiencies, achieving 1028.04  kg PCL·(mol *Aa*Cut10·h)⁻¹ and 315.96  kg PBS·(mol *Aa*Cut10·h)⁻¹, which are 20-fold and 84-fold higher than SiCut1 for PCL and PBS degradation, respectively. Furthermore, although SiCut1 exhibits significant degradation ability toward PCL, its degradation products still contain monomers and dimers; whereas after 24 h of PCL film degradation by *Aa*Cut10, only monomer products were detected, reflecting its more thorough hydrolytic capability. When polyester is present in an emulsified form, *Aa*Cut10 exhibits similarly outstanding degradation performance under the same conditions compared with previously reported enzymes. For instance, esterase from *Pseudomonas mendocina* required 24 h to clarify PBS and PCL emulsions, with only minimal turbidity reduction after 12 h (29). *Aa*Cut10 is capable of achieving degradation rates of 81.88% for PBS and 99.45% for PCL within 1 min, respectively. Furthermore, our previous studies have shown that *Aa*Cut10 effectively degrades PBAT and exhibits activity toward PHB (37). These findings collectively highlight *Aa*Cut10 as a multifunctional and highly efficient polyester-degrading enzyme with broad substrate specificity.

In addition to its remarkable polyester degradation capability, *Aa*Cut10 also exhibits outstanding properties in terms of temperature adaptability, making it highly promising for industrial applications. Most previously reported polyester-degrading enzymes—such as cutinases derived from *Fusarium solani* FSC and *Fusarium* sp. FS1301—shows optimal activity at around 50°C or higher, but their activity drops significantly under low-temperature conditions, and they generally exhibit poor thermal stability (24, 25). Moreover, although the recombinant cutinase PCC from *Paraphoma chrysanthemicola* demonstrates optimal activity at approximately 30°C, its degradation efficiency is markedly lower than that of *Aa*Cut10. Specifically, PCC requires 24 h to clarify PBS and PCL emulsions and over 60 h to cause more than 50% wt loss of PBS films (26). In contrast, *Aa*Cut10 demonstrates superior temperature adaptability and stability for polyester degradation. *Aa*Cut10 exhibits optimal degradation activity on PBS and PCL emulsions at room temperature, retains high activity even at 4°C, and maintains stability across a broad temperature range (4°C–55°C) (Fig. 3). *Aa*Cut10 also exhibits good degradation performance on PBS and PCL films at relatively low temperatures, particularly in the case of PCL films (Fig. S2). High enzyme activity at low temperatures enables lower enzyme concentrations to achieve desired degradation efficiency, effectively reducing production costs (50). These properties make *Aa*Cut10 highly suitable for low-energy industrial applications. Additionally, *Aa*Cut10 shows good compatibility with most metal ions and chemical reagents (Fig. 4) and retains degradation activity toward PBS and PCL in seawater environments (Fig. S3), further enhancing its applicability and operational flexibility under variable conditions. These advantages position *Aa*Cut10 as a robust and versatile enzyme for efficient polyester degradation under diverse industrial settings.

To investigate the reason behind the significant decrease in *Aa*Cut10’s degradation activity caused by structural changes in the active-site access channel, we conducted further analysis based on the predicted structural models. Cutinases belong to the serine hydrolase family (51). In the L209A/L216F mutant, the excessive extension of the active-site access channel may alter the spatial orientation of the substrate, thereby reducing the likelihood of effective interaction between PBS or PCL and the catalytic serine residue (Fig. 5C and D). This ultimately leads to a significant decrease in the enzyme’s degradation efficiency toward PBS and PCL. In contrast, the wild-type *Aa*Cut10 may utilize steric hindrance effects to help position the substrate in a conformation more favorable for catalysis. In addition, the L209A/L216F mutation also results in a narrower channel entrance, which may restrict the conformational freedom of PBS or PCL substrates entering the channel (Fig. 5E and F). This structural change could increase the spatial barrier that substrates must overcome to access the active site. In comparison, the wild-type *Aa*Cut10 possesses a more open binding pocket, allowing PBS or PCL substrates to more easily enter and embed into the active-site cleft in a relatively extended conformation (52). Therefore, the configuration of the substrate-binding pocket and its associated channel is a key structural feature for maintaining the high catalytic activity of *Aa*Cut10 and should be a major focus in future protein engineering efforts.

In conclusion, this study demonstrates that *Aa*Cut10 exhibits high degradation efficiency on PBS and PCL, even at low temperatures, and shows excellent compatibility with various metal ions and chemicals. These properties highlight *Aa*Cut10’s significant potential for applications in plastic biorecycling and green manufacturing, offering a sustainable solution for industrial plastic waste management.

## MATERIALS AND METHODS

### Plastics and proteins used in this study

PBS and PCL particles were purchased from Shanghai Macklin Biochemical Technology Co., Ltd. (China). The genes encoding cutinases *Aa*Cut10 (GenBank: PP116417) and *Aa*Cut4 (GenBank: PP116416), derived from the marine fungus *Alternaria alternata* FB1, were cloned into the pPICZαA vector (Invitrogen, USA) and expressed in *Komagataella pastoris* X33. The proteins were purified using HisTrap HP columns (Cytiva, USA) on an ÄKTA purifier system (Cytiva, USA), following our previously established method (37). Protein concentrations were determined using a BCA assay kit (Solarbio, China).

### Degradation assays of PBS and PCL films by *Aa*Cut10 and *Aa*Cut4

To prepare polyester films, 0.3 g of PBS or PCL particles were dissolved in 8 mL of chloroform, poured into glass Petri dishes, and allowed to solidify for 12 h to form thin films. In a low-temperature environment, heat preservation can be applied to promote film formation. For degradation assays, 5 µM of *Aa*Cut10 or *Aa*Cut4 was incubated with 50 mM Tris-HCl (pH 8.0) buffer, 500 µM Ca^2+^, and a polyester film (0.5 × 1 cm, ~2 mg) in a 1 mL reaction system. The reaction was performed at 37°C with shaking at 200 rpm, and the degradation progress was monitored and photographed at regular intervals.

### Measurement of the degradation rates of PBS and PCL films by *Aa*Cut10 and *Aa*Cut4

To determine the degradation rates of PBS and PCL films by *Aa*Cut10 or *Aa*Cut4, 5 µM of *Aa*Cut10 or *Aa*Cut4 was added to a 5 mL reaction system containing 50 mM Tris-HCl buffer (pH 8.0), 500 µM Ca^2+^, and 4–5 pieces of polyester film (0.5 × 1 cm, ~10  mg total weight). The mixture was incubated at 37 °C with shaking at 200 rpm for different periods of time, and images were taken to document the degradation process. Specifically, *Aa*Cut10 was incubated with the polyester films for 20 min, 40 min, 1 h, 1.5 h, 2 h, 3 h, and 4 h, whereas *Aa*Cut4 was incubated for 2.5 h, 3 h, 4 h, 5 h, 6 h, and 8 h. After incubation, the plastic films were carefully washed with water to terminate the reaction and then completely dried at 40°C. The remaining weight of the films was measured using an analytical balance with 0.1 mg precision (OHAUS, USA). The degradation rates of PBS or PCL plastic films by *Aa*Cut10 or *Aa*Cut4 were calculated based on the changes in weight before and after incubation. The observation of the polyester films before and after degradation by *Aa*Cut10 was performed using an inverted microscope (Nikon, Japan) at 40× magnification.

### Degradation assays of PBS and PCL emulsions by *Aa*Cut10 and *Aa*Cut4

Polyester emulsions were prepared following a previously described method with modifications (29). For PBS emulsion, 1 g of PBS particles was dissolved in 20 mL of chloroform, mixed with 100 mL of 50 mM Tris-HCl (pH 8.0) buffer, and emulsified with 0.05 g of SDS (Solarbio, China). The mixture was ultrasonicated until it transitioned from a layered oily phase to a uniform, milky liquid, and then heated and stirred for 2 h. Finally, it was diluted to 500 mL with 50 mM Tris-HCl (pH 8.0) buffer, yielding a PBS emulsion (~2 mg/mL). PCL emulsion was prepared using the same procedure. To assess the degradation activity of *Aa*Cut10 and *Aa*Cut4 on emulsions, 5 µM of enzyme was mixed with 2 mg/mL PBS or PCL emulsion at a 1:3 ratio in a 1 mL system. The reaction was manually shaken at room temperature, and turbidity changes were recorded photographically at 1 min intervals.

### Measurement of the degradation rates of emulsified PBS and PCL by *Aa*Cut10 and *Aa*Cut4

To calculate the concentration of PBS or PCL emulsion, a standard curve was generated by measuring the absorbance at 630 nm for emulsions ranging from 0 to 1.5 mg/mL. To determine the degradation rate of *Aa*Cut10 or *Aa*Cut4, 5 µM enzyme was mixed with 2 mg/mL PBS or PCL emulsion at a 1:3 ratio in a 1 mL system, yielding a final polyester concentration of 1.5 mg/mL. The absorbance at 630 nm was measured after incubation at room temperature for 0–4 min. The remaining concentration of PBS or PCL emulsion was calculated using the standard curve. The degradation rate (%) was determined as [(1.5 mg/mL − Xconcen)/1.5 mg/mL] × 100%, where “Xconcen” represents the remaining concentration of PBS or PCL emulsion. Absorbance measurements were performed using a Tecan M200PRO microplate reader (Tecan, Switzerland).

### Determination of degradation products of PBS and PCL films by *Aa*Cut10

To analyze the degradation products of polyesters, the degradation solution was processed as follows: after 24 h of incubation of PBS or PCL films with *Aa*Cut10, the reaction was terminated by boiling for 10 min. The mixture was then centrifuged at 10,625 × *g* for 10 min, and the supernatant was collected for analysis of degradation products using high-performance liquid chromatography-mass spectrometry (HPLC-MS). The analytical system consisted of an HPLC instrument (1290 Infinity, Agilent Technologies) equipped with an ACQUITY UPLC BEH C18 1.7  µm column (2.1 × 100 mm, Ireland), and an LTQ Orbitrap XL mass spectrometer (Thermo Fisher Scientific, USA). The mobile phases were solvent A (ultrapure water) and solvent B (acetonitrile). The gradient elution program was as follows: 5%–44% B (12 min), 44%–70% B (3 min), hold at 70% B (3 min), with a flow rate of 0.3 mL/min and a maximum pressure limit of 1,000 bar (49). Detection was carried out in negative ion mode with a mass-to-charge (m/z) scan range of 50–2,000.

For further analysis of PBS film degradation products, the supernatant was freeze-dried and re-dissolved in methanol, followed by gas chromatography-mass spectrometry (GC-MS) analysis. The instrument used was a GC-MS 7890B-5977B (Agilent Technologies, USA) with a 122-5532DB-5ms column (Agilent Technologies, USA; 30 m × 0.25 mm × 0.25 µm). The carrier gas flow rate was 1 mL/min. The temperature program was as follows: initial temperature at 40°C, held for 2 min; increased at 10°C/min to 300°C and held for 2 min. The inlet temperature was set at 280°C with a split ratio of 30:1. The ion source temperature was 250°C, the quadrupole temperature was 150°C, and the mass spectrometry scan range was 20–500 m/z.

### Degradation assays of PBS and PCL emulsions by *Aa*Cut10

The degradation assays of *Aa*Cut10 on PBS and PCL emulsions were conducted following a previously established method with appropriate modifications (29). Briefly, 1 µM or 0.5 µM of *Aa*Cut10 was mixed with 2 mg/mL PBS or PCL emulsion at a 1:3 ratio in a 1 mL reaction system. After incubation for 5 min, the absorbance at 630 nm was measured. The degradation activity was determined by calculating the difference in absorbance of the emulsion before and after the addition of the enzyme solution.

### Effects of temperature and pH on the degradation activity of *Aa*Cut10

To determine the optimal temperature of *Aa*Cut10 on PBS and PCL emulsions, 1 µM or 0.5 µM of *Aa*Cut10 was mixed with 2 mg/mL PBS or PCL emulsion at a 1:3 ratio in a 1 mL reaction system and incubated at various temperatures (4°C, 15°C, 23°C, 28°C, 37°C, 45°C, and 55°C) under pH 8.0 for 5 min. To determine the optimal pH, 1 µM or 0.5 µM of *Aa*Cut10 was mixed with 2 mg/mL PBS or PCL emulsion at a 1:3 ratio in a 1 mL system and incubated in 50 mM buffer at different pH levels (pH 7.0–9.0 using Tris-HCl buffer; pH 9.5-11.0 using glycine-NaOH buffer) at the optimal temperature for 5 min. The degradation activity of *Aa*Cut10 was then measured. Temperature stability was assessed by measuring the residual enzyme activity after incubating *Aa*Cut10 at different temperatures for 2 h. Similarly, pH stability was evaluated by measuring the degradation activity after storing the enzyme at various pH levels for 24 h. The highest degradation activity was defined as 100% (with temperature stability based on activity at 4°C), and the degradation activities of other samples were normalized relative to this value.

### Effects of metal ions and chemicals on the degradation activity of *Aa*Cut10

To investigate the effects of metal ions and chemicals on the degradation activity of *Aa*Cut10, different concentrations (500 µM and 10 mM) of metal ions (CuCl_2_, KCl, NaCl, MnCl_2_, CoCl_2_, MgCl_2_, FeSO_4_, ZnSO_4_, CaCl_2_, FeCl_3_, and NiCl_2_) and different concentrations (1% and 10%) of chemicals (methanol, ethanol, glycerol, Tween-80, Triton-X-100, EDTA, SDS, and β-mercaptoethanol) were added to 2 mg/mL PBS or PCL emulsion. The mixture was combined with 1 µM or 0.5 µM *Aa*Cut10 at a 3:1 ratio in a 1 mL reaction system, and the degradation activity of *Aa*Cut10 was measured after incubation for 5 min. All reactions were conducted under optimal conditions. The enzyme activity in the absence of metal ions or chemicals was set to 100% as the positive control, and the activity of *Aa*Cut10 in the presence of different metal ions or chemicals was normalized relative to the positive control.

### Orthogonal experimental design

To optimize the degradation conditions for *Aa*Cut10, an L9(3³) orthogonal experimental design was employed based on the results of single-factor experiments involving temperature, pH, and metal ions. This design was used to systematically evaluate the effects of three factors (temperature, pH, and type of metal ion) at three levels each on the relative enzymatic activity of *Aa*Cut10. When using emulsified PBS or PCL as the substrate, the tested factors and levels were as follows: temperature: 15°C, 23°C, and 28°C; pH: 7.5, 8.0, and 8.5; metal ions: 500 µM Ca^2+^ (10 mM Ca^2+^), 500 µM Mg^2+^, and 10 mM Na^+^ (10 mM Mn^2+^). Different metal ions were added to 2 mg/mL emulsified PBS or PCL and then mixed with 1 µM or 0.5 µM *Aa*Cut10 at a ratio of 3:1 to establish a 1 mL reaction system. The reactions were incubated under the designated temperature and pH conditions for 5 min, and the degradation activity was measured. The highest observed degradation activity was set as 100%, and the relative activities of other samples were normalized accordingly. The experimental data are presented as “mean ± standard deviation,” and the influence of each factor on enzymatic activity was assessed using range analysis.

### Comparison of the degradation effects of *Aa*Cut10 and its mutant on PBS and PCL

L209A/L216F is a mutant previously constructed in our laboratory based on the wild-type *Aa*Cut10 (37). Two key amino acids (Leu209 and Leu216) within the active site of *Aa*Cut10 were mutated to Ala and Phe, respectively, resulting in the L209A/L216F mutant. To evaluate the degradation activity of L209A/L216F on plastic films, 5 µM of the mutant was added to a 1 mL reaction system containing 50 mM Tris-HCl buffer (pH 8.0), 500 µM Ca^2+^, and a piece of polyester film (0.5 × 1 cm, ~2 mg). The mixture was incubated at 37°C with shaking at 200 rpm, and photographs were taken at regular intervals to monitor degradation. Then, comparing the degradation activity of the L209A/L216F mutant and wild-type *Aa*Cut10 on PBS and PCL emulsions, 2 µM or 1 µM of each enzyme was mixed with 2 mg/mL PBS or PCL emulsion at a 1:3 ratio in a 1 mL system under optimal conditions. The degradation activity was measured after incubation for 5 min. The degradation activity of the wild-type enzyme was set as 100%, and the activity of L209A/L216F was normalized relative to the wild-type *Aa*Cut10.

### Structural modeling of wild-type *Aa*Cut10 and the L209A/L216F mutant

To predict the structures of wild-type *Aa*Cut10 and its L209A/L216F mutant, we performed structure modeling using ColabFold v1.5.3 (53, 54). During structure prediction, the template_mod was set to PDB100, and the model_type was set to alphafold2_ptm. The highest-scoring structural model was selected and subjected to structure relaxation using Amber. All other parameters were set to default values.

### Statistical analysis

All data are presented as mean ± standard deviation (SD). The significance of differences was analyzed using a two-sample *t*-test with SPSS 17.0 (IBM). Differences with *P* ≤ 0.05 were considered statistically significant (*P ≤ 0.05, **P ≤ 0.01, ***P ≤ 0.001).

## Data Availability

The amino acid sequences of *Aa*Cut4 and *Aa*Cut10 have been deposited in the NCBI database with the accession numbers PP116416 and PP116417, respectively.

## SUPPLEMENTAL MATERIAL

Supplemental material aem.00833-25-s0001.pdf Figures S1 to S3; Tables S1 and S2.

ASM does not own the copyrights to Supplemental Material that may be linked to, or accessed through, an article. The authors have granted ASM a non-exclusive, world-wide license to publish the Supplemental Material files. Please contact the corresponding author directly for reuse.
