# Supplementary material for: Highly efficient degradation of polybutylene succinate (PBS) and polycaprolactone (PCL) by a recombinant marine fungal cutinase
Source: Appl Environ Microbiol. 2025 Aug 14;91(9):e00833-25. doi: 10.1128/aem.00833-25 (PMC12442398; doi:10.1128/aem.00833-25)
Supplement: Supplemental material — Figures S1 to S3; Tables S1 and S2. [file aem.00833-25-s0001.pdf]

Supplementary Results

Highly efficient degradation of polybutylene succinate (PBS) and polycaprolactone (PCL) by a recombinant marine fungal cutinase

Fengjuan Lang[a]*, Fan Fei[b,c,d]*, Chaomin Sun[b,c,d#], Shimei Wu[a#]

[a]College of Life Sciences, Qingdao University, Qingdao, China

[b]CAS and Shandong Province Key Laboratory of Experimental Marine Biology & Center of Deep Sea Research, Institute of Oceanology, Chinese Academy of Sciences, Qingdao, China

[c]Laboratory for Marine Biology and Biotechnology, Qingdao Marine Science and Technology Center, Qingdao, China

[d]College of Earth Science, University of Chinese Academy of Sciences, Beijing, China

* Fengjuan Lang and Fan Fei contributed equally to this article.

# Corresponding author

Chaomin Sun E-mail address: sunchaomin@qdio.ac.cn

Shimei Wu E-mail address: shimeiwu2016@126.com

Supplementary Figures

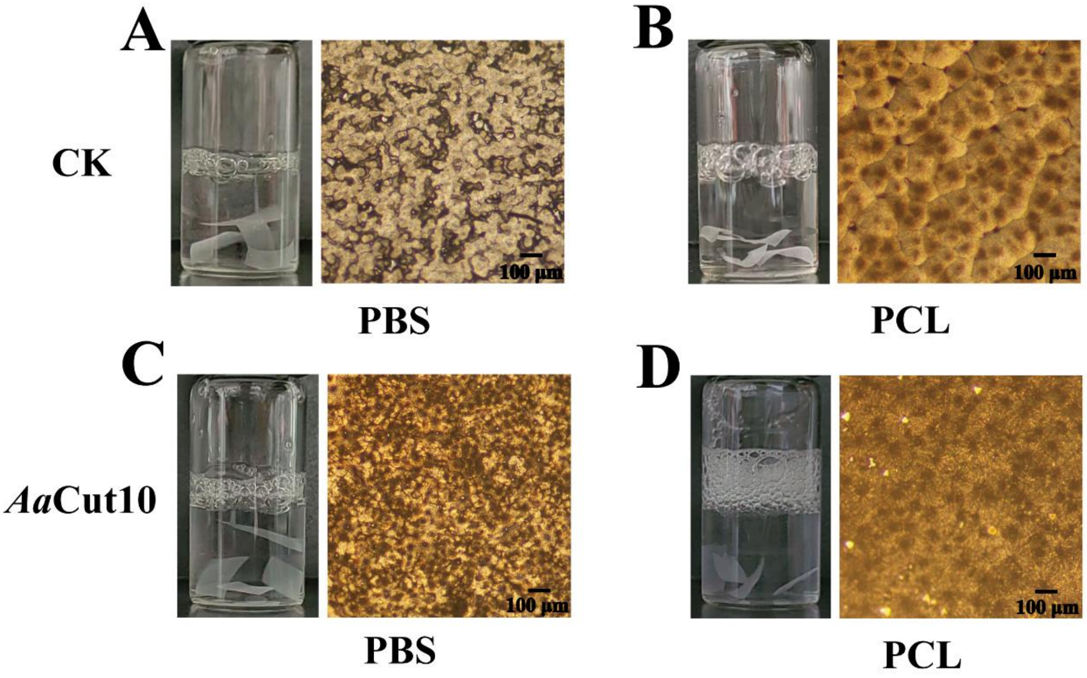

FIG S1 Macroscopic and microscopic (40×) observations of PBS and PCL films. (A, B) Macroscopic and microscopic views of untreated PBS (A) and PCL (B) films. (C, D) Macroscopic and microscopic views of PBS (C) and PCL (D) films after 20 min of degradation by *Aa*Cut10. In the assay, 5 μM *Aa*Cut10 was added to a 5 mL reaction system containing 50 mM Tris-HCl buffer (pH 8.0), 500 μM Ca^{2+}, and 4-5 polyester film pieces (0.5 × 1 cm, ~10 mg total weight), followed by incubation at 37 °C with shaking at 200 rpm. "CK" denotes the negative control group, in which the enzyme solution is replaced by an equal volume of 50 mM Tris-HCl buffer (pH 8.0). The control images correspond to the time point with the longest incubation period for each respective experimental group.

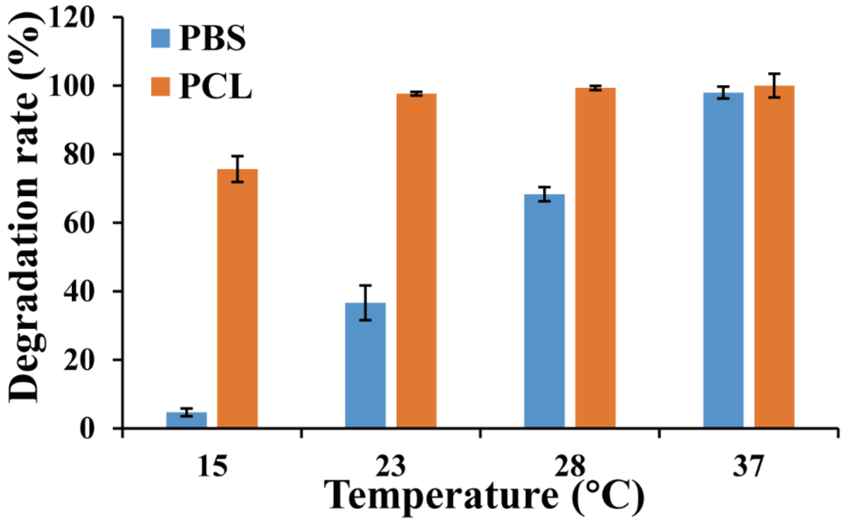

FIG S2 Degradation rates of PBS and PCL films by *Aa*Cut10 at lower temperatures (15 °C, 23 °C, 28 °C, and 37 °C). In the assay, 5 μM of *Aa*Cut10 was added to a 5 mL reaction system containing 50 mM Tris-HCl buffer (pH 8.0), 500 μM Ca^{2+}, and 4-5 polyester film pieces (0.5 × 1 cm, ~10 mg total weight), followed by incubation at 37 °C and 200 rpm for 4 h. All measurements were performed in triplicate, and the results are presented as mean ± standard deviation (n = 3).

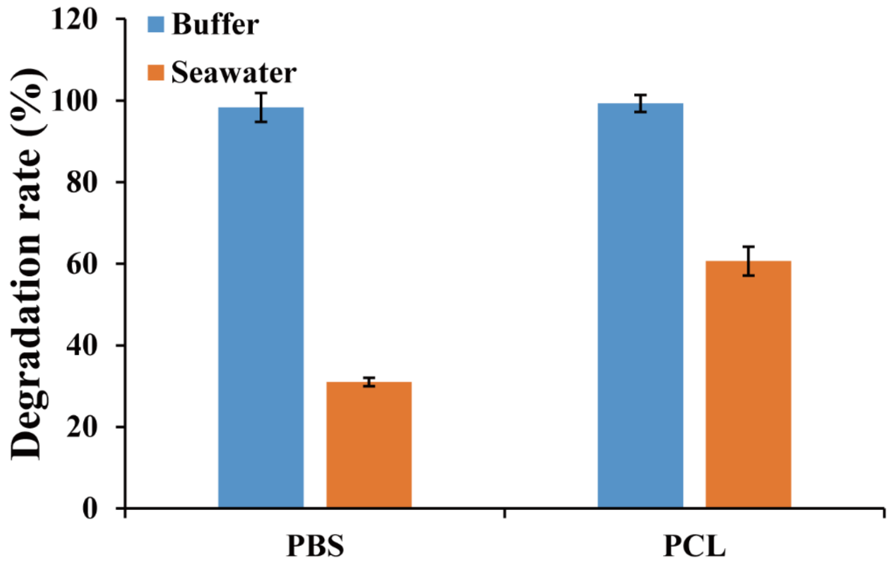

FIG S3 Degradation rates of PBS and PCL films by *Aa*Cut10 in 50 mM Tris-HCl buffer (pH 8.0) and seawater. In the assay, 5 μM of *Aa*Cut10 was added to a 5 mL reaction system containing 50 mM Tris-HCl buffer (pH 8.0), 500 μM Ca^{2+}, and 4-5 polyester film pieces (0.5 × 1 cm, ~10 mg total weight), followed by incubation at 37 °C and 200 rpm for 4 h. In the seawater systems, natural seawater was used instead of Tris-HCl buffer, and no Ca^{2+} was added. All measurements were performed in triplicate, and the results are presented as mean ± standard deviation (n = 3).

Supplementary Tables

Table S1. Orthogonal design and range analysis of factors affecting the relative degradation activity of *Aa*Cut10 toward PBS.

No.	Temperature (°C)	pH	Metal ions	Relative activity (%)
1	15	7.5	500 μM Ca^{2+}	37.66±6.21
2	15	8.0	500 μM Mg^{2+}	60.57±4.72
3	15	8.5	10 mM Na^{+}	37.02±6.75
4	23	7.5	500 μM Mg^{2+}	61.90±2.36
5	23	8.0	10 mM Na^{+}	90.07±8.25
6	23	8.5	500 μM Ca^{2+}	100.00±7.05
7	28	7.5	10 mM Na^{+}	57.71±6.08
8	28	8.0	500 μM Ca^{2+}	93.19±13.90
9	28	8.5	500 μM Mg^{2+}	95.78±2.59
k1	45.08	52.42	76.95	
k2	83.99	81.28	72.75	
k3	82.23	77.60	61.60	
R	38.91	28.86	15.35	

Note: "k" represents the average relative degradation activity of each factor (temperature, pH, metal ions) at different levels; "R" denotes the range of average relative degradation activity across levels for each factor. All measurements were performed in triplicate, and the results are presented as mean ± standard deviation (n = 3).

Table S2. Orthogonal design and range analysis of factors affecting the relative degradation activity of *Aa*Cut10 toward PCL.

No.	Temperature (°C)	pH	Metal ions	Relative activity (%)
1	15	7.5	10 mM Ca^{2+}	100.00±1.05
2	15	8.0	500 uM Mg^{2+}	28.65±0.87
3	15	8.5	10 mM Mn^{2+}	19.46±0.17
4	23	7.5	500 μM Mg^{2+}	24.37±0.70
5	23	8.0	10 mM Mn^{2+}	83.99±1.15
6	23	8.5	10 mM Ca^{2+}	69.63±1.29
7	28	7.5	10 mM Mn^{2+}	55.29±0.84
8	28	8.0	10 mM Ca^{2+}	70.53±1.12
9	28	8.5	500 μM Mg^{2+}	30.70±0.70
k1	49.37	59.89	80.05	
k2	59.33	61.06	27.91	
k3	52.17	39.93	52.91	
R	9.96	21.13	52.14	

Note: “k” represents the average relative degradation activity of each factor (temperature, pH, metal ions) at different levels; “R” denotes the range of average relative degradation activity across levels for each factor. All measurements were performed in triplicate, and the results are presented as mean ± standard deviation (n = 3).
